# β-Thymosins and Hemocyte Homeostasis in a Crustacean

**DOI:** 10.1371/journal.pone.0060974

**Published:** 2013-04-02

**Authors:** Netnapa Saelee, Chadanat Noonin, Benjamas Nupan, Kingkamon Junkunlo, Amornrat Phongdara, Xionghui Lin, Kenneth Söderhäll, Irene Söderhäll

**Affiliations:** 1 Department of Comparative Physiology, Uppsala University, Uppsala, Sweden; 2 Center for Genomics and Bioinformatics Research, Faculty of Science, Prince of Songkla University, Songkhla, Thailand; Kyushu Institute of Technology, Japan

## Abstract

Thymosin proteins are well known for their actin-binding activity. Thymosin beta 4 (Tβ4) has been associated with biological activities in tissue repair and cell migration via interaction with ATP-synthase in vertebrates, while the information of similar thymosin functions in invertebrates is limited. We have shown previously that ATP-synthase is present on the surface of crayfish hematopoietic tissue (HPT) cells, and that astakine 1 (Ast1, an invertebrate cytokine) was found to interact with this β-subunit of ATP synthase. Here, we identified five different β-thymosins from *Pacifastacus leniusculus,* designated Pl-β-thymosin1-5. The two dominant isoforms in brain, HPT and hemocytes, Pl-β-thymosin1 and 2, were chosen for functional studies. Both isoforms could bind to the β-subunit of ATP-synthase, and Pl-β-thymosin1, but not Pl-β-thymosin2, significantly increased extracellular ATP formation. Moreover, Pl-β-thymosin1 stimulated HPT cell migration in vitro and Ast1 blocked this effect. Pl-β-thymosin2 increased the circulating hemocyte number at an early stage after injection. Additionally, *in vivo* injection of Pl-β-thymosin1 resulted in significant reduction of reactive oxygen species (ROS) production in crayfish HPT whereas Pl-β-thymosin2 had a similar but transient effect. Both Pl-β-thymosins induced the expression of Ast1 and superoxide dismutase (SOD) transcripts, while silencing of endogenous Pl-β-thymosin 1 and 2 by RNAi resulted in significant reduction of the Ast1 and SOD transcripts. The diverse effects exhibited by Pl-β-thymosin1 and Pl-β-thymosin2 indicates that these proteins are involved in a complex interaction that regulates the hematopoietic stem cell proliferation and differentiation.

## Introduction

Thymosins are ubiquitous intracellular proteins and are considered as major actin sequestering proteins, which specifically binds monomeric G-actin in a 1∶1 complex. They have been shown to inhibit actin polymerization into filaments and by stabilizing actin dimers [1]. Thymosins were originally isolated from calf thymus, and subsequent studies have uncovered several thymosin types which are divided into three main groups according to their isoelectric points: α-thymosins with pI below 5.0, β-thymosins with pI between 5.0 and 7.0, and γ-thymosins with pI above 7.0 [2]. Proteins of the β-thymosin family regulate motility and actin dynamics by maintaining monomeric G actin in a nonpolymerizable form. In contrast, the first domain of the *Drosophila* Ciboulot, which is a β-thymosin, supports polymerization of actin filaments [3].

Thymosin β4 (Tβ4) is a highly conserved member of the β-thymosin family, and it is a small peptide of about 5 kDa molecular mass. This abundant peptide influences numerous cellular functions, including migration, attachment and spreading of endothelial and cancer cells [4]–[7]. Further, Tβ4 stimulates angiogenesis, cell proliferation, differentiation, wound healing and growth rate [8]–[11], prevents apoptosis and heart failure [12], as well as plays important roles in development and immune response after bacterial or viral challenge [13]–[14]. In addition, Tβ4 has been shown to be involved in cellular anti-oxidation activities because this peptide can be oxidized to sulfoxide [15] and to scavenge reactive oxygen species (ROS) that functions as signaling molecules in tumor progression [7]. Although Tβ4 is known for its intracellular activities, several studies have indicated extracellular roles for Tβ4 as well. The presence of the peptide has been demonstrated outside of cells and in blood plasma [2], indicating possible extracellular activities. Recently, it was reported that Tβ4 interacts with F_1_-F_0_ATP synthase on the plasma membrane of human umbilical vein endothelial cells (HUVECs), and that interaction resulted in an increase of the extracellular ATP concentration, which led to an increase in cell migration [16]. In comparison to the vast amount of information that is present for Tβ4 in vertebrates, the knowledge of thymosin-like proteins from invertebrates is fairly limited. Most studies deal with *Drosophila* Ciboulot, a 14.4 kDa protein that contains three thymosin domains, and plays a major role in axonal growth during brain metamorphosis [17]. Another interesting invertebrate member of the β-thymosin family is thypedin a protein that is involved in foot regeneration in Hydra (Cnidaria) and which contains 27 copies of a β-thymosin-like domain [18].

In a previous study, we have demonstrated that a β-subunit of F_1_ATP synthase may function as a receptor for astakine 1 (Ast1), a hematopoietic cytokine, and that this receptor is present on the surface of a small subpopulation of the crayfish HPT cells. This interaction inhibits extracellular ATP formation [19]. In this work we have found that Ast1 affects the expression of some β-thymosin-like transcripts, and we characterized two crayfish thymosin isoforms that also interacted with the β-subunit of F_1_ATP synthase. The β-subunit of F_1_ATP synthase mediated interaction between Ast1 and crayfish thymosins. In addition, our results show that these β-thymosins may play a role in hematopoiesis by promoting cell proliferation and differentiation.

## Materials and Methods

### Experimental animals

Freshwater crayfish (*P. leniusculus*) were purchased from lake Hjälmaren, and lake Vättern, Sweden. The crayfish were maintained in tanks with running aerated water at 10°C. Only healthy and intermolt animals were used in the experiments.

### Hematopoietic tissue (HPT) cell culture

The HPT cells were isolated from freshwater crayfish as described previously by Söderhäll *et al* [19] with minor modifications. Briefly, the hematopoietic tissue was dissected from the dorsal side of the stomach, washed with crayfish phosphate-buffered saline (CPBS; 10 mM Na_2_HPO_4_, 10 mM KH_2_PO_4_, 150 mM NaCl, 10 mM CaCl_2_ and 10 mM MnCl_2_, pH 6.8), and then incubated in 700 µl of 0.1% collagenase Type I and Type IV (Sigma) in CPBS at room temperature (RT) for 40 min. The isolated cells were collected by centrifugation at 3000 × g for 5 min at RT to remove the collagenase solution. The cells were washed twice with 1 ml CPBS and the undigested tissue was removed. The isolated HPT cells were then resuspended in modified L-15 culture medium [19] and subsequently seeded in 96 well plates at a density of 8×10^4^ cells/150 µl. After about 30 min of attachment, the cells were supplemented with 3 µl cell-free crayfish plasma, and the culture plates were incubated at 16°C. One-third of the medium was changed every second day.

### Identifying of spliced variants of β-thymosin and their expression in different tissues

A suppression subtractive hybridization (SSH) experiment was performed earlier [20] to identify genes in the HPT that were affected by the addition of Ast1. Among the up regulated transcripts we could identify several β-thymosin-like sequences. After cDNA cloning using forward primer: 5'- ACTTCCTGCTCACATTTTATCG -3' and reverse primer 5'- AAACATTTTGGCTTGCAGAACT -3' five different transcripts encoding five alternatively spliced variants of β-thymosin were detected and sequenced.

The BLAST algorithm (http://www.ncbi.nlm.nih.gov/blast) was used to analyze the cDNA sequence and the Expert Protein Analysis System (http://www.expasy.org/) to analyze the amino acid sequence of the β-thymosin. The amino acid sequences of β-thymosins from *P. leniusculus* were aligned to human Tβ4 (accession no. NP_066932.1) using multiple sequence alignment created with ClustalX 2.1.

In order to determine the distribution of the β-thymosin transcripts in different tissues, total RNA was extracted from different crayfish tissues, including eye stalk, heart, hepatopancreas, HPT, hemocytes, intestine, nerve cord, and testis by using the Gene Elute Total Mammalian RNA extraction kit (Sigma-Aldrich), followed by RNase free DNase I (Ambion) treatment. Equal amounts of total RNA was used for cDNA synthesis with ThermoScript (Invitrogen) according to the manufacturer’s instructions and analyzed for expression of β-thymosin by RT-PCR, using the following primers: 5'- ACTTGCCTAAGGTCGACACTG -3′ and 5'- CTTCCTTAGTAGGGAGTTTGCAC -3'. The PCR program used was as follows: 94°C, 2 min, followed by 30 cycles of 94°C for 30 s, 60°C for 30 s, and 72°C for 40 s, and the transcription of a 40S ribosomal protein was used as an internal control. All PCR products were analyzed on 1.5% agarose gel stained with GelRed.

### Recombinant protein expression and purification

Total RNA was isolated from crayfish HPT using Trizol LS (Invitrogen), followed by DNase treatment and cDNA synthesis as described above. The open reading frames (ORF) encoding two β-thymosins (Pl-β-thymosin1 and Pl-β-thymosin2) were amplified using the synthesized forward primer (5'-GATCGGATCCAGCACCGAGGCCGCAATCAAGGAC-3') and the reverse primer (5'-GACTCTCGAGTTAGGCTTTCTTCTCCAGCTCAATC-3'). The resulting PCR products were then cloned into the pGEX4T-1 bacterial expression vector at BamHI and XhoI cleavage sites to generate glutathione S-transferase (GST)-fused β-thymosin proteins. The generated pGEX4T-1-thymosins constructs were confirmed by DNA sequencing to correspond to Pl-β-thymosin1 and Pl-β-thymosin2.

*Escherichia coli* strain BL21 (DE3) containing recombinant pGEX4T-1-Pl-β-thymosin1, pGEX4T-1-Pl-β-thymosin2 or control pGEX4T-1 plasmids were grown under vigorous shaking at 37°C until the *A*_600_ reached 0.6. IPTG (1 mM) was added to induce the expression and the bacteria were cultured for additional 4 h at 37°C. The collected bacterial pellets were suspended in PBS buffer (140 mM NaCl, 2.7 mM KCl, 10 mM Na_2_HPO_4_, 1.8 mM KH_2_PO_4_, pH∶7.3) and lysed by sonication at 4°C for a few seconds. The cell lysate was centrifuged at 13,000 g for 15 min at 4°C to collect the supernatant. The soluble recombinant proteins were purified using glutathione Sepharose 4B (GE Healthcare) according to the manufacturer’s recommendations. The purified proteins were analyzed by 12.5% SDS-PAGE.

### GST pull down assay

Freshly prepared HPT was homogenized in 500 µl radioimmunoprecipitation assay buffer (RIPA buffer) (50 mM Tris, 150 mM NaCl, 10 mM EDTA, 1% NP–40, 0.1% SDS, pH 7.5), containing 1x protease inhibitor cocktail (Roche Diagnostics, Germany). The tissue lysate was then centrifuged at 13,000 rpm for 20 min at 4°C, and the resulting whole tissue lysate was used in a GST pull down assay.

Recombinant GST-Pl-β-thymosin1, GST-Pl-β-thymosin2 or GST as a control, were immobilized on glutathione-Sepharose beads respectively and mixed with HPT lysate and incubated in PBS binding buffer (137 mM NaCl, 2.7 mM KCl, 8 mM Na_2_HPO_4_, 1.46 mM KH_2_PO_4_) overnight at 4°C. The beads were extensively washed with PBS, and the bound proteins were dissolved in Laemmli sample buffer, separated by 12% SDS-PAGE and finally analyzed by western blot.

### Western blot analysis

Protein samples were dissolved in Laemmli sample buffer (62.5 mM Tris-HCl, 2% SDS, 10% (v/v) glycerol, 0.1 M DTT, 0.01% bromophenol blue, pH 6.8) and separated using a 12.5% SDS-PAGE and then electro-transferred onto a polyvinylidene fluoride membrane (PVDF) (Bio-Rad, America) for 2 h. The blot was blocked subsequently in Tris buffered saline (TBST) (0.5% Tween 20 in 20 mM Tris-HCl, 150 mM NaCl, pH 7.5) containing 5% skim milk for 1 h. The membrane was incubated with anti-ATP synthase β subunit (Santa Cruz Biotechnology ) (dilution 1∶3000) or anti-Ast1 (dilution 1∶1000) as previously described by Lin et al [19] or anti-Tβ4 (Tβ-4 (FL-44): sc-67114, Santa Cruz Biotechnology)(dilution 1:7000) in blocking buffer for 1 h at RT. After extensive washing, the membrane was incubated with a secondary antibody conjugated with horseradish peroxidase (Sigma) (dilution 1∶7,500), and detection was performed using the ECL western blotting reagent kit (Amersham Biosciences) according to the manufacturer’s instructions.

### Detection of secreted Pl-β-thymosin and endogenous protein in hemocytes and HPT

Hemolymph was collected from crayfish and centrifuged at 1 000 x g for 5 min at 4 °C. The resulting supernatant was designated as plasma and was separated from the hemocyte pellet. The plasma was then subjected to ultracentrifugation at 130 000 x g for 2 h at 4 °C to remove hemocyanin which is an abundant protein in the plasma. The supernatant plasma after ultracentrifugation was collected and the proteins were precipitated by acetone. The resulting protein pellet was dissolved in PBS, and the protein concentration was determined. The hemocyte pellet and isolated HPT cells were homogenized and the protein concentrations in the cell lysates were determined. The obtained proteins from both tissues and plasma were then subjected to SDS-PAGE (100 µg protein for each sample) and western blotting using antibody against Tβ4 (Tβ-4 (FL-44): sc-67114, Santa Cruz Biotechnology).

In addition, the secretion of Pl-β-thymosins from cultured HPT cells was also investigated. The HPT cells were isolated and cultured in 96-well plates as described above. Since expressions of Pl-β-thymosins were found to be induced by Ast1, recombinant Ast1 was supplemented into the culture medium (final concentration 200 nM). Then the cells were maintained at 16°C, and 24 h after Ast1 treatment, the culture medium was collected and centrifuged at 1 000 x g for 5 min. The cell-free conditioned medium was collected and subjected to acetone precipitation. The protein pellet was dissolved in SDS-sample buffer and analyzed by western blotting as above.

### Assay of extracellular ATP formation

Extracellular ATP formation was assayed as described previously by Lin et al [19] with minor modifications. HPT cells (3×10^5^) were seeded into a 20-mm^2^ part near the edge of a tissue culture dish (35 m×100 mm), and after 16 h incubation the cells were washed with 200 μl Hepes buffer (10 mM Hepes, 150 mM NaCl, pH 7.4), and incubated with 150 µl reaction buffer (Hepes buffer containing 100 µM ADP, 20 mM potassium phosphate and 2 mM MgCl_2_) for 3 min. Then 10 µl samples were taken for extracellular ATP determination by using ATP Biomass Kit HS (BioThema) and the light emission was detected by a Luminometer (LKB Wallac 1250) according to the manufacturer’s instruction. The formation of ATP was used as the individual control value for each dish. Then the cells were washed with Hepes buffer, incubated with recombinant GST-Pl-β-thymosin1 or GST-Pl-β-thymosin2 or GST control protein (100, 200 or 400 nM) for 30 min prior to addition of the reaction buffer and used for the determination of extracellular ATP. The formation of ATP was calculated by comparison with each individual control value.

### Circulating hemocyte count

Crayfish were injected at the base of a walking leg with GST-Pl-β-thymosin1, GST-Pl-β-thymosin2 or GST as a control as described previously [21]. The injected dose was 5 pmol/g crayfish fresh weight (n  =  5–8 for each group). After injection, the crayfish were kept in tanks with aerated running water at 10°C. After 6 and 18 h, blood samples were taken, and the total hemocyte number was determined. Hemocyte index was calculated as the number of hemocytes after injection divided by the number of hemocytes before injection.

### Detection of reactive oxygen species (ROS) in vivo

To examine effect of the Pl-β-thymosins on ROS level in HPT, crayfish (n  =  4–5 for each group) were injected at the base of a walking leg with GST-Pl-β-thymosin1, GST-Pl-β-thymosin2 or GST at 5 pmol/g fresh weight of the crayfish. After injection the crayfish were kept in tanks with aerated running water at 10°C as described above. At 6, 18, and 24 h after injection, the HPTs were dissected from crayfish. The fresh tissues were put in 24-well plate and washed three times with PBS. ROS production was measured by incubating the fresh tissue with 2 ml of 5 µg/ml of nonfluorescent 2',7'-dichlorofluorescin diacetate (DCF-DA) (Sigma) for 10 min at RT in darkness. This compound is membrane permeable and it is intracellularly deacetylated to form green fluorescent product. After 10-min incubation, the tissues were washed 4 times with PBS and transferred to 96-well plate containing PBS. The fluorescence intensity was then immediately determined using a microplate reader with excitation wavelength of 485 nm and emission wavelength of 535 nm. The results were reported as % fluorescent intensity comparing to the GST control.

### Cell migration assay

To assay for stimulation of cell migration, BD Falcon^™^ cell culture insert with polyethylene terephthalate (PET) track-etched membranes (0.8 µm pore size) (BD Biosciences) was used. The 24-well culture plates were filled with 0.9 ml of L-15 medium containing purified GST control (200 nM) or recombinant Pl-β-thymosins (200 nM) with/without Ast1 (200 nM) and the cell culture inserts were placed in each well. Then suspended HPT cells were seeded on the PET membrane at a concentration of 1.2×10^5^ cells/0.35 ml. After 18 h, the inserts were moved to new wells containing fresh medium mixed with purified proteins. Five hours later, non-migrated cells on the upper side of the membrane were removed by scraping using a cotton tip. The migrated cells attached to the lower side of the membrane were counted under a microscope. All data were obtained from 3–5 independent experiments.

### In vitro treatment of recombinant Pl- β-thymosins

Two hundred nM (final concentration) of purified GST-Pl-β-thymosin1, GST-Pl-β-thymosin2 or GST were added to culture HPT cells every second day. After 1 week, total RNA was extracted from HPT cells, and RT-PCR was performed to analyze Ast1 and SOD mRNA expression. The following primers were used: for Ast1, 5'-ATGCGAGGAGTTAGTGTG- 3' and 5'-CTAGTAGTAGGAGTCGAGCGTGTTGTC'- 3'; for SOD, 5'-ATGGTGAACATGACTCTCCC-3' and 5'-GTTGTACGTCCTCTGGTACTG-3'. All PCR products were then analysed on 1% an agarose gel, stained with GelRed.

### RNA interference

For RNA silencing, double-stranded RNA (dsRNA) was produced using oligonucleotide primers for GFP and *Pl*-β-thymosins designed to append with a T7 promoter (italics) at the 5' terminal: for dsGFP : 5'-*TAATACGACTCACTATAGGG*CGACGTAAACGGCCACAAGT-3' and 5'-*TAATACGACTCACTATAGGG*TTCTTGTACAGCTCGTCCATGC-3' ; for dsPl-β-thymosin : 5'*TAATACGACTCACTATAGGG*ATGAGCACCGAGGCCGCAAT- 3' and 5'- *TAATACGACTCACTATAGGG*AGGGCAGAAGGATTAAATCC-3' ; for dsPl-β-thymosin2 : 5'-*TAATACGACTCACTATAGGG*CGTGGAGCAGGAGAAGCAACA -3' and 5'-*TAATACGACTCACTATAGGG*TCTTGACTAGTAGGGAGGACG-3'. To generate dsRNA, 1 µg PCR product was purified by gel extraction (Qiagen) and used as a template for in vitro transcription by using Megascript kit (Ambion) according to the manufacturer's instruction.

Transfection of dsRNA into the HPT cells was performed as described by Lin et al [22] with minor modifications. Briefly, 8 µl dsRNA (250 ng/µl) was mixed with 3 µl calf histone H2A (histone from calf thymus, type II-A, 1 mg/ml dissolved in modified L-15 medium) (Sigma-Aldrich) and incubated for 5–10 min at RT and then mixed with 20 µl cell culture medium before adding into one well with HPT cells (96-well plates). The cells were then incubated at 16°C. One third of the total volume of medium was changed every second day during incubation of the HPT cell cultures. After 7-days of incubation, total RNA was extracted from the HPT cells to determine RNAi efficiencies. The HPT cells were washed twice with CPBS, and then total RNA was extracted to monitor the transcription of Ast1, mannose binding lectin (MBL), superoxide dismutase (SOD) and kazal proteinase inhibitor (KPI) by semi-quantitative RT-PCR.

### LPS injection

Crayfish (N  =  3) were injected with 50 µg LPS and kept in tanks with aerated water. At 6 and 24 h after injection, plasma, hemocytes and HPT were collected from the crayfish. Protein samples were prepared from each tissue as described above and then subjected to SDS-PAGE and western blotting to examine levels of Pl-β-thymosin protein.

## Results

### P. leniusculus expresses β-thymosins with one to five β-thymosin domains

**Figure 1:**
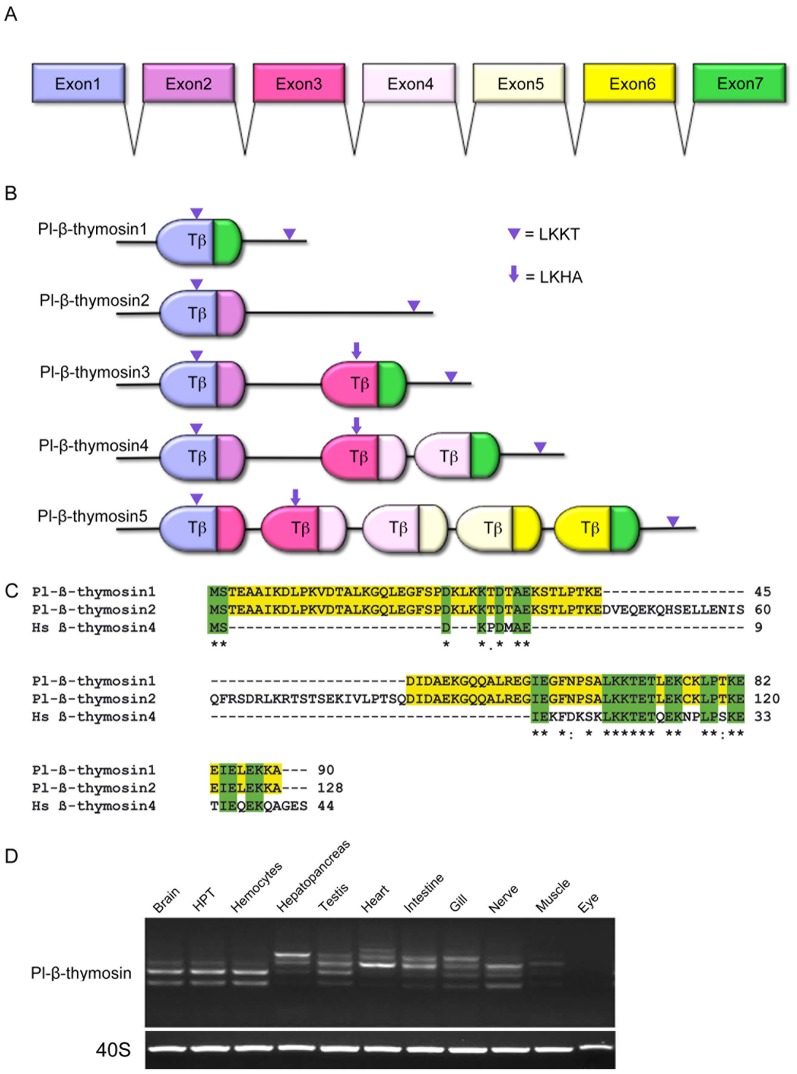
At least 5 β-thymosins are expressed in *P. leniusculus.* A) Putative exon structure of a β-thymosin gene in *P. leniusculus* determined by sequencing five different spliced transcripts. B) Protein structure determined for Pl-β-thymosin1–5. The typical Tβ domains (pfam 01290) are encoded for by different exons (as indicated by color that reflects the encoding exons in A) and the different actin binding motifs are indicated by triangles (LKKT) and arrows (LKHA). C) Sequence alignments of Pl-β-thymosins with human Tβ4 (accession no. NP_066932.1). Consensus sequences are highlighted with green background. D) Tissue distribution of different Pl-β-thymosins in various tissues of *P. leniusculus* detected by RT-PCR. The mRNA expression of 40S ribosomal protein is shown as an internal control.

In order to find genes in the HPT that are influenced by Ast1, we used a PCR-based cDNA subtraction in a previous study [20]. After screening of the forward SSH cDNA library, we could identify a protein named crustacean hematopoietic factor (CHF) involved in preventing apoptosis, as we have described earlier [20]. Furthermore, we could identify partial sequences of several transcripts with similarity to human Tβ4, and here we describe the characterization of some of these transcripts. We cloned five cDNAs of β-thymosin and named these as Pl-β-thymosin1–5 (GenBank Accession number JX272322 – JX272325 for Pl-β-thymosin1-4, and KC460336 for Pl-β-thymosin5, Figure S1). These different Pl-β-thymosins are most likely the result of alternative splicing of a gene with the putative exon structure as described in Figure 1A. Structure analysis of the different thymosin-like transcripts revealed that Pl-β-thymosin1 and Pl-β-thymosin2 contains one β-thymosin domain (Tβ in Figure 1B; pfam 01290) encoded for by exons 1+7, and exons 1+2 respectively. The putative structure of Pl-β-thymosin3 contains two Tβ domains, Pl-β-thymosin4 is composed of three Tβ domains, and finally Pl-β-thymosin5 contains five Tβ domains. The consensus actin-binding motif LKKT was identified in the N-terminal Tβ domain of all isoforms, whereas the second Tβ domain of Pl-β-thymosin3-5 in addition contains an actin-interacting motif (LKHA) that is more similar to *Drosophila* Ciboulot (LKNA, and LKHT) [23]. Moreover, a LKKT actin-binding motif was detected in the C-terminal part of Pl-β-thymosin1-5 transcripts outside of the typical Tβ domains (Figure 1B). As is the case for Tβ4 in human, the Pl-β-thymosins have no signal peptides, and most likely are localized to the cytosol. A BLASTX search showed that the Pl-β-thymosins share high similarity with thymosins from other crustaceans, mainly to the related crayfish species *Procambarus clarkii* (Accession no GU937433) and *Cherax quadricarinatus* (Accession no JF284580), and the crab *Eriocheir sinensis* (Accession no FJ372906). Further, the Tβ domains of Pl-β-thymosins contain the highly conserved amino acids found in human Tβ4 (Figure 1C) but also with *Drosophila* Cibuolot. Apart from the conserved Leu_16_-Lys_17_ of the actin binding motif (LKKT/LKHT), Phe_11,_ the first of five amino acids of the so called linker [23] that connects an N-terminal helix with the LKKT motif is conserved in all *P. leniusculus* Tβ domains. Furthermore, Thr_21_, Glu_23_, Lys_24_, Leu_27_ and Pro_28_ are conserved from crayfish to vertebrates [24], as are Lys_30_-Glu_31_ and Glu_36_-Lys_37_ of the C-terminal helix (Figure 1C).

An interesting observation is that most invertebrate β-thymosins does contain more than one Tβ domain, where *Hydra* thypedin represents an extreme with 27 Tβ domains. In contrast, most vertebrate β-thymosins is single Tβ domain proteins. However, we could demonstrate that Pl-β-thymosin1-2 are expressed as a single Tβ domain protein as is shown in Figure 1B. The shortest sequence Pl-β-thymosin1 was detected mainly in brain, HPT, hemocytes, and nerve tissues, and the faint band of this size, detected in the other tissues, may be due to infiltrating hemocytes (Figure 1D). By using primers designed to exon 1 and 7 (Figure 1A) we were able to identify five different Pl-β-thymosins in several tissues (Figure S1) and the expression profile showed a clear dominance for Pl-β-thymosin1 and 2 in brain, HPT and hemocytes, and therefore this study is focused on these two transcripts. In contrast, Pl-β-thymosin4 was highly expressed in hepatopancreas, and Pl-β-thymosin3 and 4 were both found in intestine, gills, testis and heart while Pl-β-thymosin5 expression was detected exclusively in heart.

### Pl-β-thymosin1 and 2 can bind to an ATP synthase β-subunit

To investigate further putative functions of Pl-β-thymosin1 and 2 in hematopoiesis, we produced recombinant GST-Pl-β-thymosin1 and GST-Pl-β-thymosin2 fusion proteins of 35.9 kDa and 40.1 kDa, respectively, in *E. coli* BL21 using the pGEX4T-1 plasmid (containing a GST tag of 26 kDa). Both recombinant proteins were soluble and used in binding studies (Figure S2).

**Figure 2:**
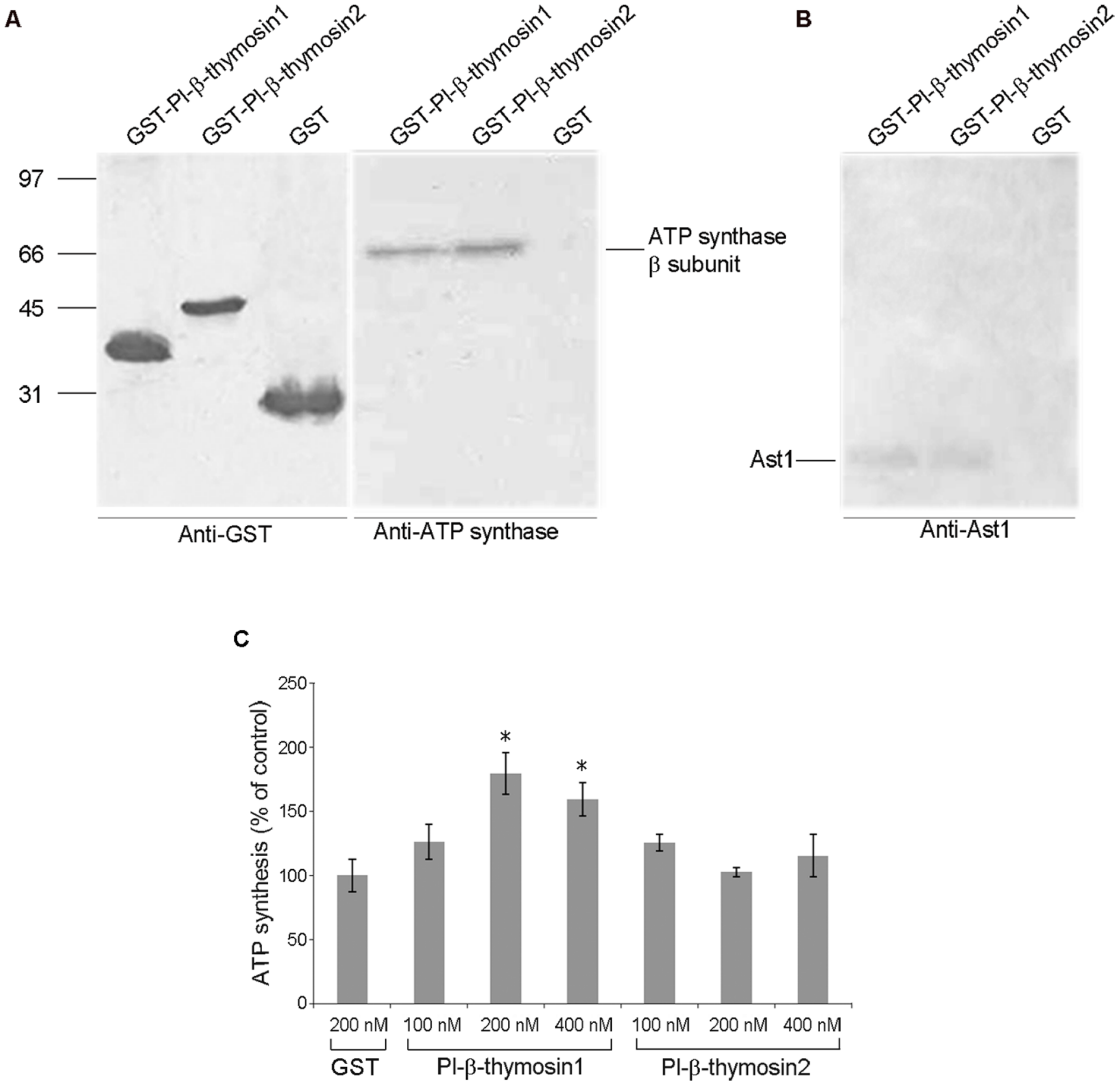
Pl-β-thymosins interact with the β-subunit of ATP synthase. A) Protein-protein interaction of Pl-β-thymosins and the β-subunit of ATP synthase detected by a GST pull down assay. GST-Pl-β-thymosin1, GST-Pl-β-thymosin2 or GST (control) were used as baits for proteins in a HPT lysate. The bound proteins were eluted and detected by western blot analysis using anti-ATP synthase β-subunit antibody. B) Protein-protein interaction of Pl-β-thymosins and Ast1 detected by a GST pull down assay using recombinant proteins and HPT lysate as described in (A). The bound proteins were eluted and detected by western blot analysis using anti-Ast1 antibody. C) Effect of recombinant Pl-β-thymosin1 and Pl-β-thymosin2 on extracellular ATP synthesis in HPT cells. The columns represent the mean of three separate experiments, and error bars represent SE values. * P < 0.05 when compared to GST control group.

We have previously shown that the hematopoietic cytokine Ast1 could bind to ATP-synthase present on a subpopulation of HPT stem cells [19]. Since human Tβ4 recently was found to interact with the β-subunit of ATP-synthase present on the surface of human vein endothelial cells (HUVECs) [16], we used a pull-down assay to examine whether any of these Pl-β-thymosins could form a complex with the β-subunit of ATP synthase. As shown in Figure 2A, the pull-down assays revealed that the β-subunit of F_1_ATP synthase in a HPT lysate, coprecipitated with GST-Pl-β-thymosin1 and GST-Pl-β-thymosin2 but not with the GST alone. Further, we could show that Ast1 was bound together with the Pl-β-thymosin1 or 2 and the β-subunit of F_1_ATP synthase (Figure 2B). However, no direct interaction between Ast1 and the two Pl-β-thymosins could be found indicating that these proteins bind to different sites on the β-subunit of F_1_ATP synthase.

ATP synthase is present on the surface of some HPT cells and the binding of Ast1 to this surface enzyme resulted in a block of the ATP formation [19]. Thus, we next assessed the role of Pl-β-thymosins on extracellular ATP formation in the HPT cells. Then, purified recombinant GST-Pl-β-thymosin1, GST-Pl-β-thymosin2 and GST control proteins were used in this assay. The results showed that the recombinant GST-Pl-β-thymosin1 (at concentrations of 200 and 400 nM) was able to increase extracellular ATP synthesis in HPT cells (Figure 2C) whereas no significant effect on ATP synthesis was detected for GST-Pl-β-thymosin2 or the GST control.

**Figure 3:**
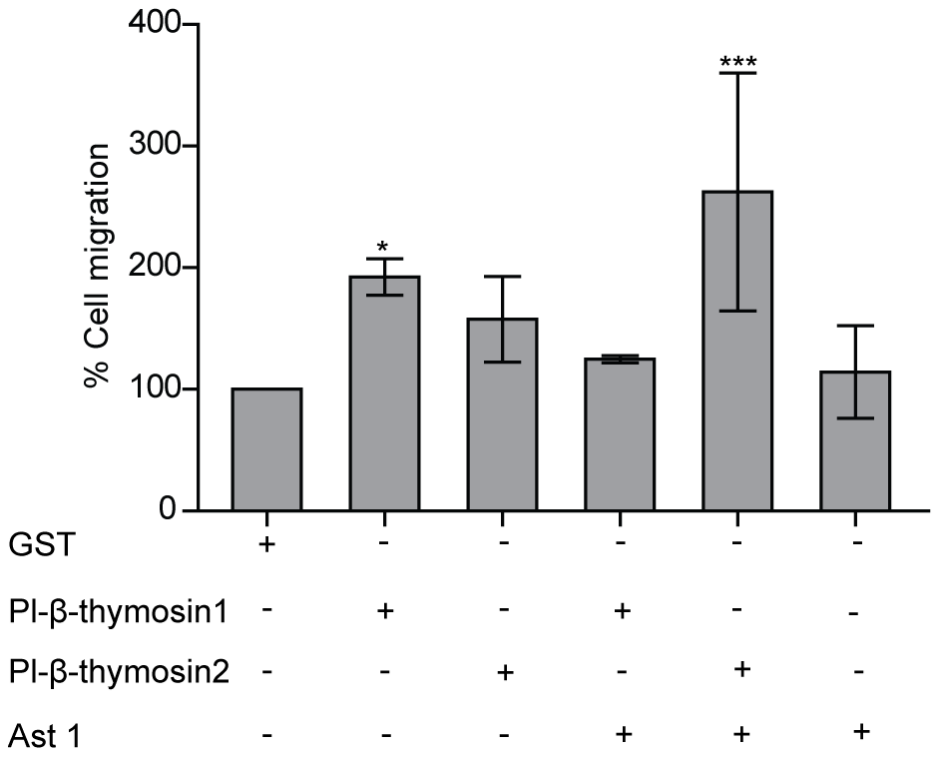
HPT cell migration is affected by Pl-β-thymosin1 and Pl-β-thymosin2. The isolated HPT cells were cultured in a culture chamber containing polyethylene terephthalate (PET) track-etched membranes. The cells were incubated with indicated recombinant proteins and the cells migrated to the bottom side of the membrane were counted. Pl-β-thymosin1 (200 nM) promoted HPT cells migration and Ast1 (200 nM) could diminish the effect of Pl-β-thymosin1, and together with Pl-β-thymosin2 induced migration. The columns represent the mean of 3–5 independent experiments, and error bars represent SE values. * P < 0.05, *** P < 0.001 when compared to GST control group.

Binding of human Tβ4 to ATP-synthase was shown to stimulate HUVEC migration, and since Ast1 and the Pl-β-thymosins likewise could bind to this enzyme we decided to test the effect of these proteins on HPT cell migration in a transwell assay. Similar to human β-thymosin, Pl-β-thymosin1 significantly stimulated cell migration while a significant effect could not be observed for Pl-β-thymosin2 or Ast1 (Figure 3). Interestingly, Ast1 could efficiently block the migration response of Pl-β-thymosin1 while it enhanced the cell migration effect of Pl-β-thymosin2.

### Exogenous Pl-β-thymosins stimulate a transient increase in semigranular cell (SGC) number

**Figure 4:**
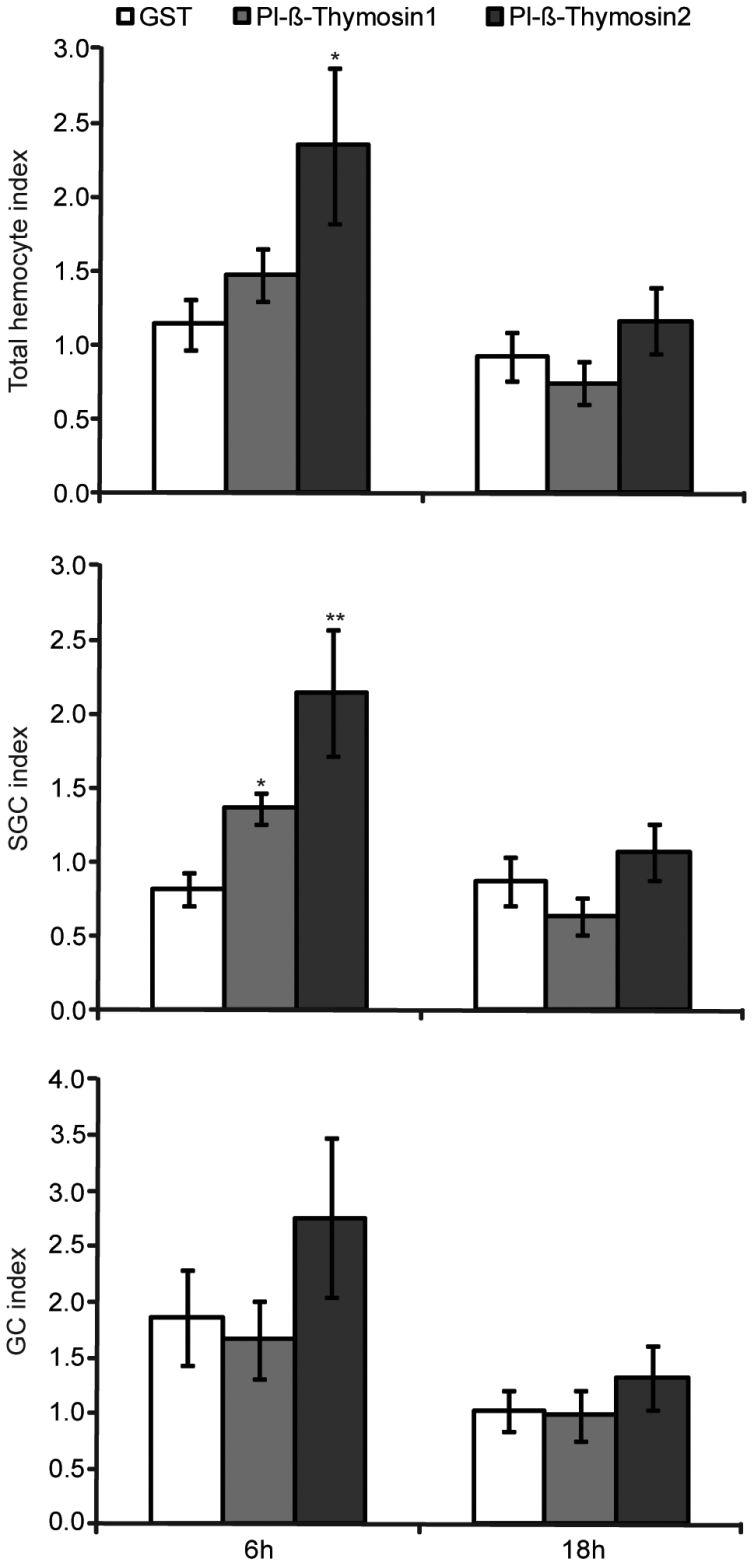
Pl-β-thymosin2 induces a transient increase in circulating hemocyte number. Total and differential hemocyte number (semigranular and granular cells) at 6 h and 18 h post injection of Pl-β-thymosin1 or Pl-β-thymosin2 or GST (5 pmol/g crayfish weight) were examined. Hemocyte number was counted and divided by the number before injection. Five to eight animals were used in each experimental group, and different animals were used for 6h and 18h. The columns represent the mean of 5–8 animals, and error bars represent SE values. * P < 0.05, ** P < 0.01 when compared GST control.

We could detect Pl-β-thymosins as upregulated transcripts in a SSH library of Ast1 treated cultured HPT cells. Since Ast1 is also known to induce proliferation of HPT cells as well as differentiation and release of new hemocytes into the circulation we decided to investigate if Pl-β-thymosin1 or Pl-β-thymosin2 had any impact on hemocyte number. Ast1 injection into live crayfish results in an increased number of circulating hemocytes [21], while silencing of Ast1 clearly blocks new hemocyte release from the HPT [25]. Silencing of Pl-β-thymosins *in vivo* was not possible due to their abundance in many tissues as opposed to Ast1, which is restricted to hemocytes and nerves. Ast1 is also a secreted protein, whereas Pl-β-thymosins most likely are intracellular proteins. However, β-thymosins are frequently detected in human plasma and are recognized as extracellular regulators in a variety of different processes [16], [26]. Thus, we tested the effect of Pl-β-thymosin1 and Pl-β-thymosin2 injection on hemocyte number. As shown in Figure 4, Pl-β-thymosin2 had a clear but transient effect (6 h but not 18 h after injection) on the total number of circulating hemocytes and in particular the SGC. In contrast, Pl-β-thymosin1 had no significant effect on total hemocyte number but significantly increased SGC at the same time point.

### Pl-β-thymosins affect ROS production in different ways

**Figure 5:**
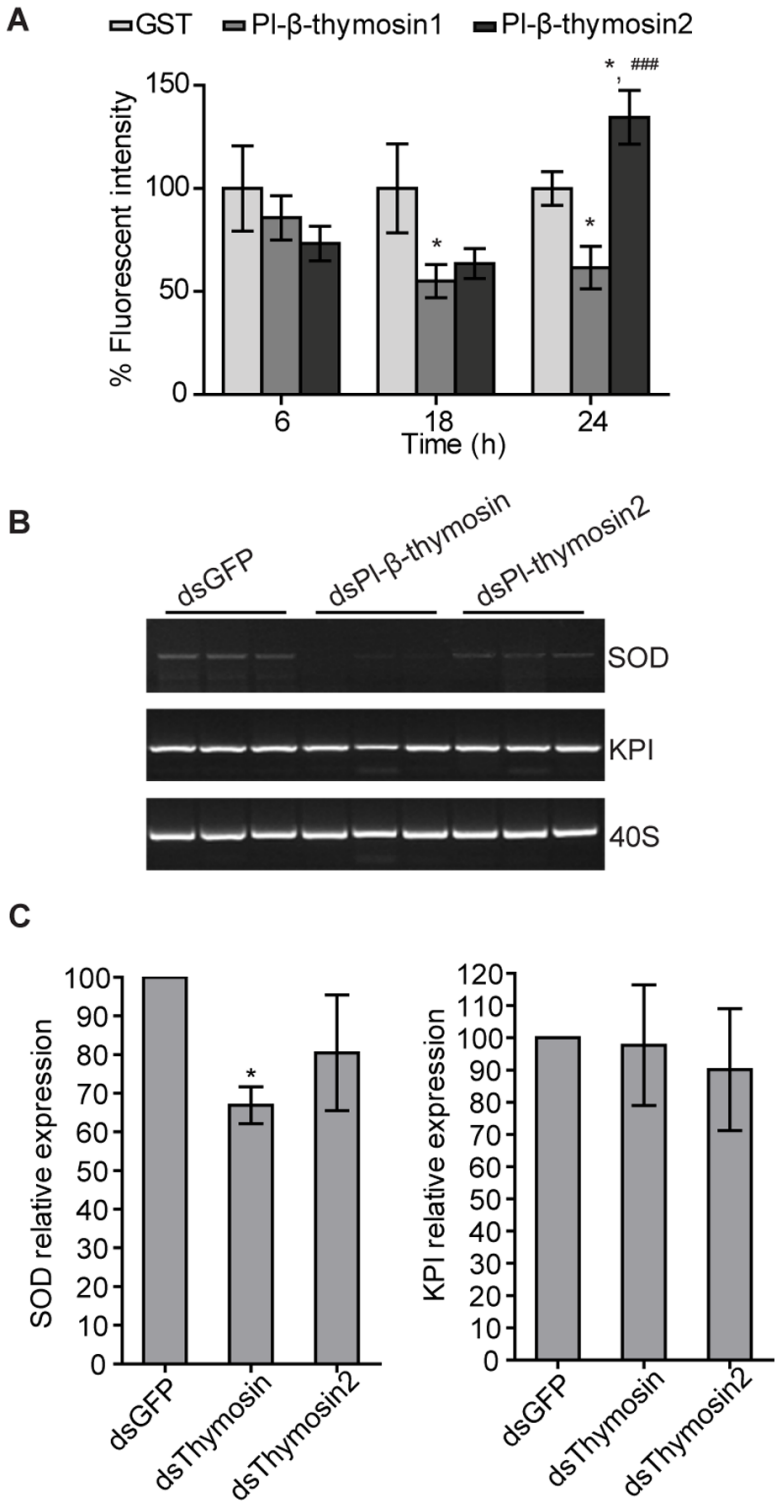
Two Pl-β-thymosins are involved in oxidative stress in different ways. A) *In vivo* effect of Pl-β-thymosin1 or Pl-β-thymosin2 on ROS level in the APC. Recombinant Pl-β-thymosin1, Pl-β-thymosin2 or GST were injected into crayfish. At 6, 18 and 24 h after injection the HPT were dissected for ROS level detection. At 18 h, both Pl-β-thymosins caused reduction of ROS level. This reduction of ROS was stable at 24 h for Pl-β-thymosin1 injection, but transient for Pl-β-thymosin2. Four animals were used in each experimental group. * P < 0.05when compared to GST control, and ^###^ P < 0.001 when compared to Pl-β-thymosin1 injected group. B-C) Suppression of SOD expression after Pl-β-thymosin RNAi in HPT cells. After transfection with dsPl-β-thymosin or dsPl-β-thymosin2, total RNA was extracted from HPT cells to determine RNAi efficiencies with specific primer for Pl-β-thymosin and Pl-β-thymosin2 (Figure S3). The transcription levels of SOD, KPI and 40S ribosomal protein mRNA were assayed by RT-PCR (B) or qPCR (C). This experiment was repeated three times with similar result. * P < 0.05 when compared to dsGFP control (C).

A specific area, named the APC (anterior proliferative centre) in the anterior part of crayfish HPT is known to be highly proliferative, and to produce reactive oxygen species (ROS) [27]. This high ROS activity is further increased if microbial polysaccharides are injected into a crayfish and after this injection hemocytes are released from the HPT. Since, Pl-β-thymosins showed a transient effect on the circulating hemocyte number, we tested if extracellular Pl-β-thymosins may be involved not only in ATP formation but also in ROS production in HPT. At 6 h post-injection, there was no significant difference in the ROS level between the GST control and Pl-β-thymosin groups, but a significant decrease of ROS was found at 18 h after Pl-β-thymosin1 injection (Figure 5A). Although, Pl-β-thymosin2 also caused some reduction of ROS at 18 h but this difference was not statistically significant. Interestingly, the two Pl-β-thymosins tested showed opposite effects on ROS production at 24 h post-injection. While injection of recombinant Pl-β-thymosin1 resulted in a statistically significant reduction of ROS production in HPT, when compared to the GST injected animals (Figure 5A), the injection of Pl-β-thymosin2 instead caused significant induction of ROS compared to the control.

Oxidative stress and accumulation of ROS in the APC appear to stimulate hemocyte differentiation and release, but later also an increased apoptosis in the HPT. ROS detoxification is taken care of by superoxide dismutase (SOD) and thus, we decided to investigate the effect of both Pl-β-thymosins on SOD expression, by gene silencing via RNAi. Cultured HPT cells were treated with either dsRNA of Pl-β-thymosins (dsPl-β-thymosin and dsPl-β-thymosin2) or GFP (dsGFP). While dsPl-β-thymosin did silence both forms but as shown in Figure S3 Pl-β-thymosin1 was more efficiently silenced, and dsPl-β-thymosin2 completely silenced Pl-β-thymosin2. Seven days after dsRNA treatment, the transcription of SOD and KPI was monitored by semi-quantitative RT-PCR (Figure 5B) and qPCR (Figure 5C). Interestingly, a down regulation of the SOD transcript was observed after both dsRNA treatments with a stronger effect found after dsβ-thymosin treatment. These results may imply that endogenous Pl-β-thymosins, and in particular Pl-β-thymosin1 is of importance for SOD expression. However, our data also demonstrate that addition of extracellular of both Pl-β-thymosins is sufficient to induce SOD gene expression in HPT cells (Figure S4).

### LPS injection and Ast1 treatment affect the extracellular and intracellular level of Pl-β-thymosin proteins in hemocytes, plasma and HPT cells

Since the results obtained above suggest that Pl-β-thymosins may be involved in hemocyte homeostasis, we investigated the protein level of Pl-β-thymosins in plasma, hemocytes and HPT cells in response to LPS-induced hemocyte loss. This treatment mimics an infection and results in an increase of Ast1 protein in plasma [25] and a rapid loss of hemocytes followed by an increase due to synthesis and release of new hemocytes from the HPT [28].

**Figure 6:**
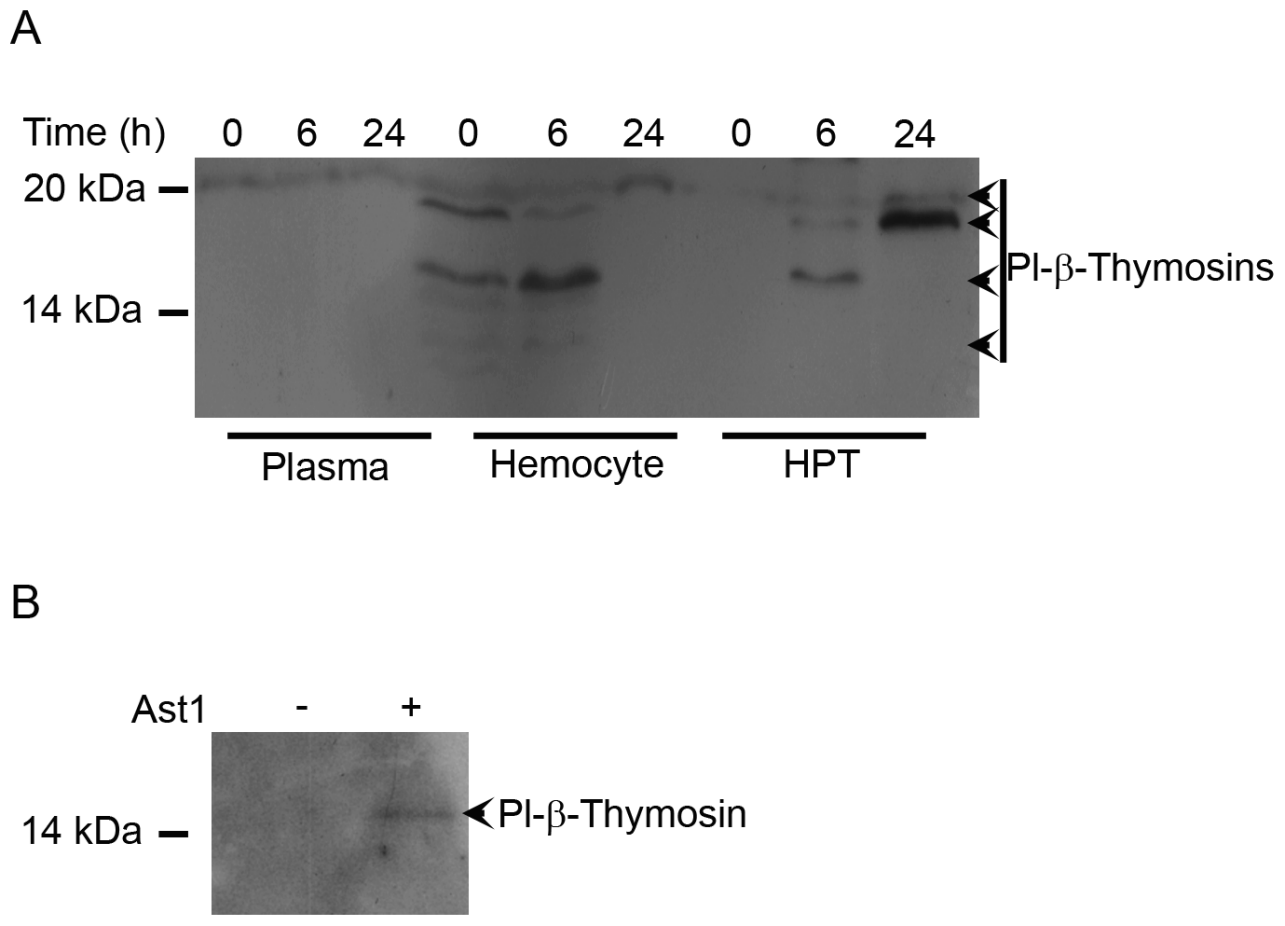
Protein level of Pl-β-thymosins after LPS injection and Ast1 treatment. A) Crayfish were injected with LPS (50 µg) to induce hemocyte loss and the new hemocyte synthesis and release. At 6 and 24 h after injection, plasma, hemocytes and HPT were collected, and the protein level of Pl-β-thymosins was examined. Three crayfish were used in this experiment and the same results were observed in each crayfish. B) HPT cells were isolated and cultured in the absence or presence of Ast1 (200 nM). At 24 h after Ast1 treatment, the culture medium was collected and secretion of Pl-β-thymosins from the HPT cells into the culture medium was investigated. This experiment was repeated 3 times.

The result in Figure 6A shows that β-thymosins can be detected in plasma, although at fairly low levels, and more interestingly the levels of different β-thymosins seem to increase in hemocytes as well as in HPT cells after an injection with LPS.

The presence of Pl-β-thymosins in plasma indicates that these proteins or at least some of the types can be secreted, and, when HPT cells were isolated and cultured in the presence or absence of Ast1, secretion of a Pl-β-thymosin from the cultured cells was clearly detected at 24 h after treatment with Ast1 (Figure 6B).

## Discussion

The β-thymosins are abundant peptides in vertebrates and invertebrates, but not in prokaryotes or yeast [29]. Mammalian Tβ4 is the most common and most studied member of the β-thymosin family. This peptide is highly expressed in most cell types and the intracellular concentration in leukocytes is more than 300 µM [29]. Tβ4 has important intracellular functions mainly due to its ability to bind to monomeric (G-) actin. However, these peptides are also present in extracellular fluids such as blood serum, and Tβ4 has been shown to act as an extracellular modulator in tissue repair, cell migration and immune defense [30]. Nearly all known vertebrate β-thymosins are small peptides, which contain only one single conserved β-thymosin domain (Tβ domain), whereas the invertebrates usually produce multi-Tβ domain proteins, among which *Drosophila* Cibulout and *Hydra* Thypedin are the most well-known [17], [18], [29].

Here, we describe two single Tβ domain proteins that are highly expressed in the HPT, brain and hemocytes of the invertebrate, *P. leniusculus* and report some of their extracellular and intracellular effects in hemocyte behavior and homeostasis. We could identify at least five different β-thymosins, all of which probably is derived from alternative splicing of the same gene sequence. The different transcripts are differentially expressed in various tissues, where Pl-β-thymosin1 and Pl-β-thymosin2 are the most abundant in brain, HPT and hemocytes. These two transcripts consists of one Tβ domain, but differ in length and moreover in the sequence of the Tβ domain C-terminal helix which previously have been shown to be very important for the actin-binding function of this protein [3]. These are one of the few single Tβ domain proteins described in invertebrates. Proteins that contain a Tβ domain are known to regulate actin polymerization and do so in different ways. Either they are actin sequestering and thereby block polymerization as is the case for Tβ4, or they promote actin assembly and thereby have an impact on cell mobility. The Glu_35_-Gln_36_ in the Tβ domain of Tβ4 was shown to be necessary for its actin sequestering activity [3], and corresponding amino acids were present in Pl-β-thymosin2, while Pl-β-thymosin1 instead had Asp-Ala at this position. This difference may explain some of the different activities recorded for these two proteins in our study. Moreover, the sequence of the so-called linker region, consisting of 5 amino acids immediately followed by the actin binding motif LKKT differ from that in Tβ4, and this may indicate a weaker actin interaction [23].

**Figure 7:**
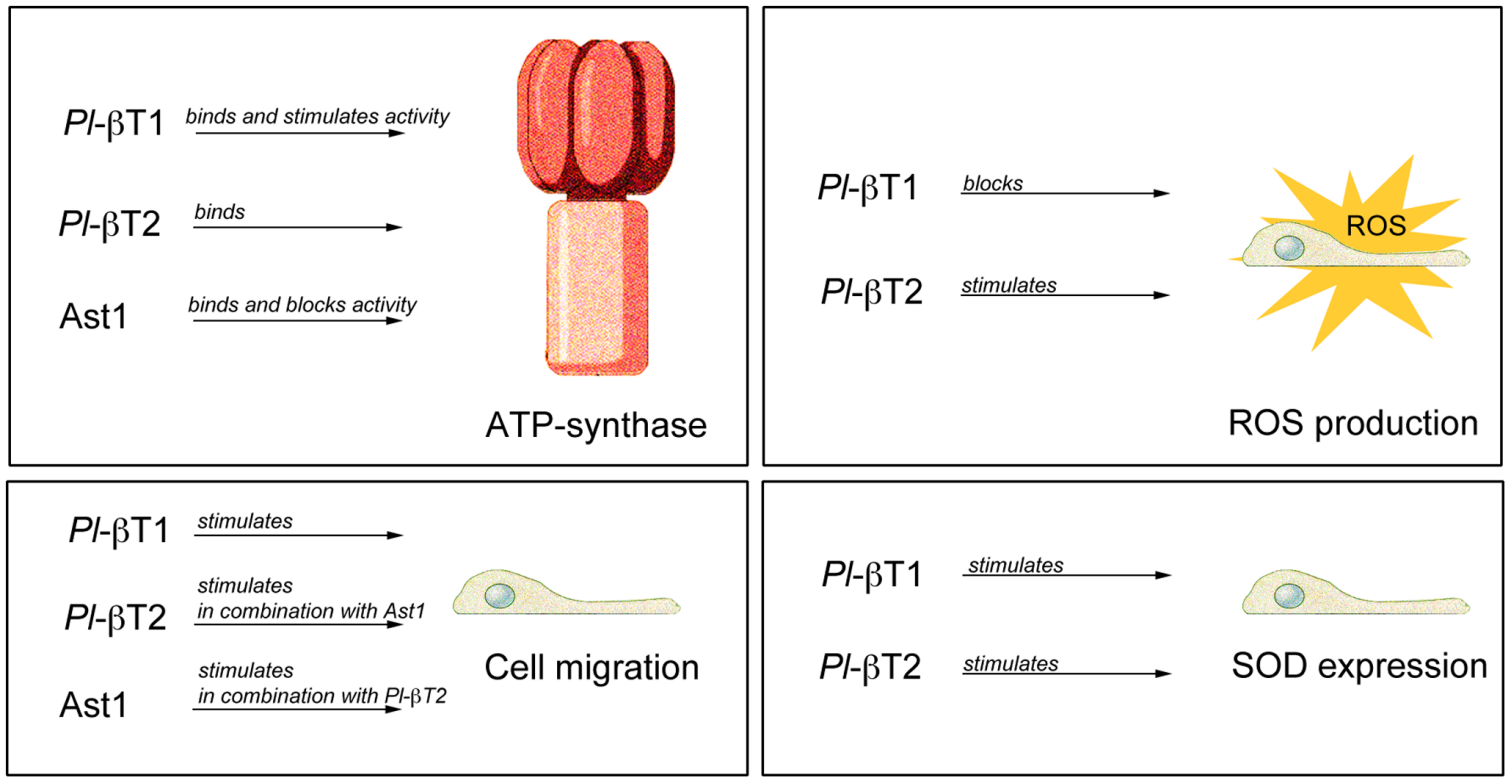
Summary of the effects of Ast1 and Pl-β-thymosins in crayfish. A brief summary of the main findings of this study on the effects of Pl-β-thymosin1 and Pl-β-thymosin2 on crayfish hematopoietic stem cells. A) Both Pl-β-thymosins bind to ATP-synthase as does Ast1, and Pl-β-thymosin1 stimulates the activity of ATP-synthase. B) Pl-β-thymosin1 stimulates cell migration, while Pl-β-thymosin2 only stimulates migration when combined with Ast1. C) Pl-β-thymosin1 blocks ROS production in the hematopoietic tissue, while Pl-β-thymosin2 stimulates this activity. D) Both Pl-β-thymosin1 and Pl-β-thymosin2 stimulates SOD mRNA expression, and their knockdown results in lower SOD expression.

Apart from intracellular actin binding activities, β-thymosins have several extracellular activities [29]. Extracellular administration of Tβ4 supports wound healing, anti-inflammatory responses and promotes migration of a number of different cell types into wounds [2], [7], [9], [11]. Although Tβ4, as well as the β-thymosins described in the present study seem to be synthesized as intracellular proteins and not secreted, it seems as if these proteins in a hitherto unknown way are released from cells into the surrounding milieu. The level in crayfish plasma detected by a Tβ4 antibody was very low, and it is possible that this may be due to occasional cell rupture in the hemolymph. However, in vitro cultured HPT cells could be induced to release β-thymosin by treatment with Ast1. Further, in similarity with earlier studies, we could also show extracellular effects of β-thymosins on cell migration as well as of hemocyte release from the HPT (Figure 7). HUVECs migration required an interaction of Tβ4 with F_1_-F_0_ATP synthase on the outside of the cell membrane [16]. Similar to Tβ4, both Pl-β-thymosin1 and Pl-β-thymosin2 were found to interact with the β-subunit of F_1_ATP synthase. However, only Pl-β-thymosin1 could interfere with ATP production resulting in an increased synthesis of ATP (Figure 7). The reason for this could be that Pl-β-thymosin1 is likely to have a structure more similar to Tβ4, which was shown to share structural characteristics with the ATP synthase inhibitory factor 1 (IF1). In addition, we have previously shown that Ast1 cytokine interacts with the β-subunit of F_1_ATP synthase present on the cell surface of some HPT cells [19]. Therefore, by their ability to bind to F_1_ATP synthase, Ast1 and the extracellular Pl-β-thymosins may be involved in regulating extracellular ATP signaling, but in different ways. This is also in agreement with our result that, crayfish Pl-β-thymosin1 could promote migration of HPT cells in an *in vitro* assay, and this effect was blocked by Ast1 addition.

Apart from stimulating cell migration, the Pl-β-thymosins also affected ROS production in the APC of the hematopoietic tissue. Interestingly, the actin cytoskeleton has been implicated in regulating ROS release from mitochondria, and thereby acts as a modulator of apoptosis and/or differentiation [31]. Extracellular Tβ4 did reduce intracellular ROS in cardiac fibroblasts [26], and in our hands Pl-β-thymosin1 had a similar effect in decreasing ROS activity of the APC (Figure 7). In contrast, the longer protein Pl-β-thymosin2 had an opposite effect and stimulated ROS activity and Pl-β-thymosin2 also had a transient but clearly significant effect on the number of circulating hemocytes. After a rapid increase in hemocyte number it falls again and it is possible that a rapid fall in circulating hemocyte number may stimulate new synthesis and higher ROS activity in the APC as is the case after LPS stimulation [27]. In addition to affect ROS production, both Pl-β- thymosins could increase the expression of the antioxidant enzyme SOD, and an intracellular effect on SOD was also confirmed by gene silencing of Pl-β-thymosins. However, silencing of Pl-β-thymosin2 slightly affected SOD expression when compared to silencing of Pl-β-thymosin1, again showing opposite roles for these two Pl-β-thymosins in regulating ROS (Figure 7). Their actin binding abilities may differ due to the differences in important amino acids of the Tβ domain, and whether these differences also have implication for their different roles in ATP production and ROS production needs to be further clarified. A microbial infection or LPS injection, which mimics a bacterial infection, is known to cause a dramatic reduction of circulating hemocytes and a significant increase of plasma Ast1 level [25]. The high concentration of plasma protein Ast1 induces cell proliferation in HPT, which is found to be significantly increased after LPS injection [27]. In addition, as shown here this increase in plasma Ast1 may induce the production and secretion of the Pl-β-thymosins which then resulted in migration or release of differentiated HPT cells from the tissue. Therefore, the results in this study indicate a corporation between Pl-β-thymosins and Ast1 in regulating crayfish hemocyte homeostasis. The diverse effects exhibited by Pl-β-thymosin1 and Pl-β-thymosin2 indicate that these proteins are involved in a complex interaction that regulates the hematopoietic stem cell proliferation and differentiation.

## Supporting Information

Figure S1 **β-thymosins in** ***P. leniusculus.*** Amino acid sequence alignment of five different Pl-β-thymosins from *P. leniusculus*.
(PDF)

Figure S2 **The expression and purification of recombinant Pl-β-thymosins were analyzed by 12.5% SDS-PAGE.** Lane M, protein molecular weight markers; lane 1 and 2, the expression of the GST-Pl-β-thymosin1 before and after induction; lane 3, the purified GST-Pl-β-thymosin1; lane 4 and 5, the expression of GST-Pl-β-thymosin2 before and after induction; lane 6, the purified Pl-β-thymosin2.
(TIF)

Figure S3 **Pl-β-thymosins RNAi HPT cells.** After tranfection with dsPl-β-thymosin, dsPl-β-thymosins2 or dsGFP, the HPT cells were harvested for RNA extraction to determine RNAi efficiency with specific primer for Pl-β-thymosin and Pl-β-thymosin2.
(TIF)

Figure S4 **Pl-β thymosin treatment enhances SOD mRNA expression in HPT cells.** SOD mRNA were analyzed by RT-PCR at seven days after treatment with GST-Pl-β-thymosin1, GST-Pl-β-thymosin2 or GST control protein. A 40S ribosomal gene was used as internal control. This experiment was performed three times with similar result.
(TIF)
