# Supplementary material for: β-Thymosins and Hemocyte Homeostasis in a Crustacean
Source: PLoS One. 2013 Apr 2;8(4):e60974. doi: 10.1371/journal.pone.0060974 (PMC3614969; doi:10.1371/journal.pone.0060974)
Supplement: Figure S1 — β-thymosins in P. leniusculus. Amino acid sequence alignment of five different Pl-β-thymosins from P. leniusculus. (PDF) [file pone.0060974.s001.pdf]

```
Pl-β-T1        MSTEAAIKDLPKVDTALKGQLEGFSPDKLKKTDTAEKSTLPTKE----------------
Pl-β-T2        MSTEAAIKDLPKVDTALKGQLEGFSPDKLKKTDTAEKSTLPTKEDVEQEKQHSELLENIS
Pl-β-T3        MSTEAAIKDLPKVDTALKGQLEGFSPDKLKKTDTAEKSTLPTKEDVEQEKQHSELLENIS
Pl-β-T4        MSTEAAIKDLPKVDTALKGQLEGFSPDKLKKTDTAEKSTLPTKEDVEQEKQHSELLENIS
Pl-β-T5        MSTEAAIKDLPKVDTALKGQLEGFSPDKLKKTDTAEKSTLPNKE----------------
               ********************************************

Pl-β-T1        ------------------------------------------------------------
Pl-β-T2        QFRSDRLKRTSTSEKIVLPTSQ--------------------------------------
Pl-β-T3        QFRSDRLKRTSTSEKIVLPTSQDVEAEKQAKAHLEAVEGFNSANLKHANTQEKIVLPAKE
Pl-β-T4        QFRSDRLKRTSTSEKIVLPTSQDVEAEKQAKAHLEAVEGFNSANLKHANTQEKIVLPAKE
Pl-β-T5        ----------------------DVEAEKQAKAHLEAVEGFNSANLKHANTQVKIVLPAKE

Pl-β-T1        ------------------------------------------------------------
Pl-β-T2        ------------------------------------------------------------
Pl-β-T3        ------------------------------------------------------------
Pl-β-T4        DIETEKTHQSIFQGVTAFDKSQMRHAETEEKVALPAKE----------------------
Pl-β-T5        DIETERTHQSIFQGVTGFDKASMRHAETQEKIALPTKEDNETEKTHQSIFQGVMAFDKSQ

Pl-β-T1        ------------------------------------------------------DIDAEK
Pl-β-T2        ------------------------------------------------------DIDAEK
Pl-β-T3        ------------------------------------------------------DIDAEK
Pl-β-T4        ------------------------------------------------------DIDAEK
Pl-β-T5        MRHAETEEKVSLPAKEDIVTERAHQGIFQRLVSFDRSEMKHADTTEKNVLPSKSDIDAEK
                                                                     ******

Pl-β-T1        GQQALREGIEGFNPSALKKTETLEKCKLPTKEEIELEKKA
Pl-β-T2        GQQALREGIEGFNPSALKKTETLEKCKLPTKEEIELEKKA
Pl-β-T3        GQQALREGIEGFNPSALKKTETLEKCKLPTKEEIELEKKA
Pl-β-T4        GQQALREGIEGFNPSALKKTETLEKCKLPTKEEIELEKKA
Pl-β-T5        GQQALREGIEGFNPSALKKAETLEKCKLPTKEEIELEKKA
               ****************************************
```
