# Supplementary figures and images for: β-Thymosins and Hemocyte Homeostasis in a Crustacean
Source: PLoS One. 2013 Apr 2;8(4):e60974. doi: 10.1371/journal.pone.0060974 (PMC3614969; doi:10.1371/journal.pone.0060974)

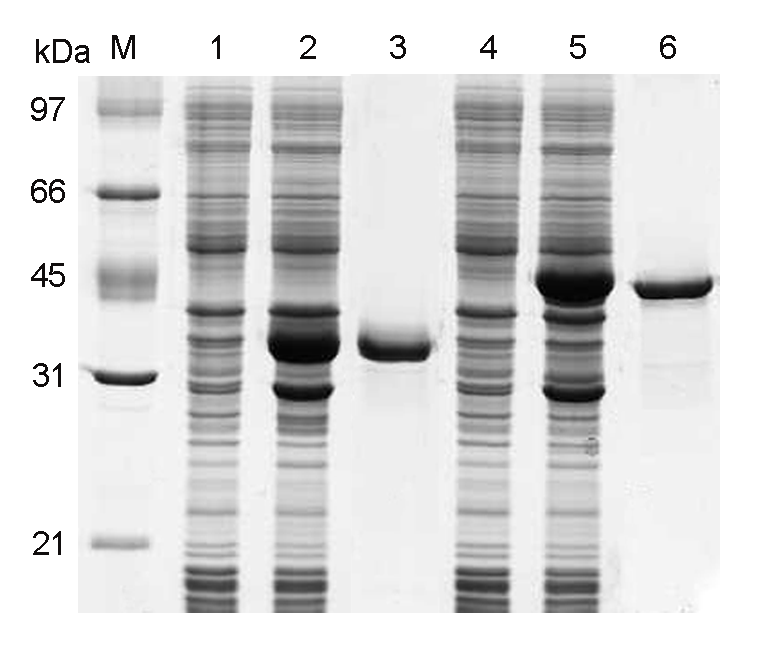

Supplement: Figure S2 — The expression and purification of recombinant Pl-β-thymosins were analyzed by 12.5% SDS-PAGE. Lane M, protein molecular weight markers; lane 1 and 2, the expression of the GST-Pl-β-thymosin1 before and after induction; lane 3, the purified GST-Pl-β-thymosin1; lane 4 and 5, the expression of GST-Pl-β-thymosin2 before and after induction; lane 6, the purified Pl-β-thymosin2. (TIF) [file pone.0060974.s002.tif]

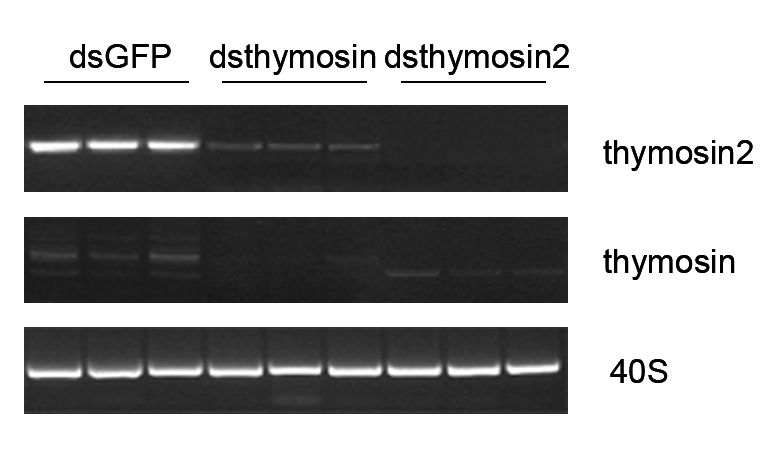

Supplement: Figure S3 — Pl-β-thymosins RNAi HPT cells. After tranfection with dsPl-β-thymosin, dsPl-β-thymosins2 or dsGFP, the HPT cells were harvested for RNA extraction to determine RNAi efficiency with specific primer for Pl-β-thymosin and Pl-β-thymosin2. (TIF) [file pone.0060974.s003.tif]

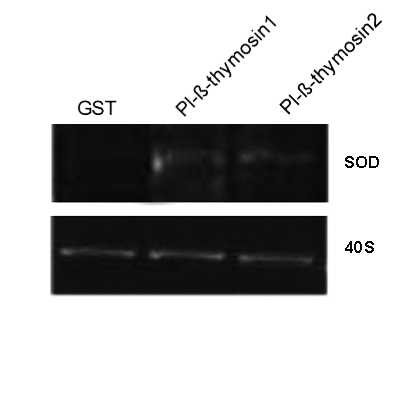

Supplement: Figure S4 — Pl-β thymosin treatment enhances SOD mRNA expression in HPT cells. SOD mRNA were analyzed by RT-PCR at seven days after treatment with GST-Pl-β-thymosin1, GST-Pl-β-thymosin2 or GST control protein. A 40S ribosomal gene was used as internal control. This experiment was performed three times with similar result. (TIF) [file pone.0060974.s004.tif]
